# Reactive optical matter: light-induced motility in electrodynamically asymmetric nanoscale scatterers

**DOI:** 10.1038/s41377-018-0105-y

**Published:** 2018-12-12

**Authors:** Yuval Yifat, Delphine Coursault, Curtis W. Peterson, John Parker, Ying Bao, Stephen K. Gray, Stuart A. Rice, Norbert F. Scherer

**Affiliations:** 1 James Franck Institute, The University of Chicago, 929 E. 57th Street, Chicago, IL 60637 USA; 2 Department of Chemistry, The University of Chicago, 929 East 57th Street, Chicago, IL 60637 USA; 3 Department of Physics, The University of Chicago, 929 East 57th Street, Chicago, IL 60637 USA; 4 Center for Nanoscale Materials, Argonne National Laboratory, 9700 South Cass Avenue, Argonne, IL 60439 USA; 5 Present Address: Université Bordeaux, CNRS LOMA, UMR 5798, F-33400 Talence, France; 6 Department of Chemistry, Western Washington University, 516 High Street, Bellingham, WA 98225 USA

From Newton’s third law, which is known as the principle of *actio et reactio*^1^, we expect the forces between interacting particles to be equal and opposite for closed systems. Otherwise, “nonreciprocal” forces can arise.^2^ This has been shown theoretically in the interaction between dissimilar optically trapped particles that are mediated by an external field.^3^ As a result, despite the incident external field not having a transverse component of momentum, the particle pair experiences a force in a direction that is transverse to the light propagation direction.^3,4^ In this letter, we directly measure the net nonreciprocal forces in electrodynamically interacting asymmetric nanoparticle dimers and nanoparticle structures that are illuminated by plane waves and confined to pseudo one-dimensional geometries. We show via electrodynamic theory and simulations that interparticle interactions cause asymmetric scattering from heterodimers. Therefore, the putative nonreciprocal forces are actually a consequence of momentum conservation. Our study demonstrates that asymmetric scatterers exhibit directed motion due to the breakdown of mirror symmetry in their electrodynamic interactions with external fields.

The development of light-driven nanomotors, which are devices that convert light energy into autonomous motion, has attracted tremendous interest.^5^ Various optical methods can produce rotational motion^6^ or, using primarily photoreactive materials, translational motion.^7^ A promising direction toward creating such nanomotors has arisen from recent theoretical work, which predicted that dissimilar particles that are illuminated by an electromagnetic plane wave will experience a “nonreciprocal” net force.^3,4^ This autonomous motion occurs in the absence of an applied external driving force in the transverse plane, but the transverse motion of a particle pair arises in reaction to its asymmetric scattering. Simulations demonstrated that these nonreciprocal forces vary with interparticle separation. However, there has not been a direct and straightforward experimental demonstration of this phenomenon.

In this letter, we experimentally demonstrate this optical self-motility phenomenon with optically bound dimers of dissimilar-size metallic nanoparticles (NPs), thereby rectifying the deficiency. Our experimental findings are quantitatively supported by electrodynamic simulations. In addition, we demonstrate optical self-motility beyond particle pairs by generating and measuring the translational motion of asymmetrical nanoparticle assemblies.

Our experiments were performed using a standard optical trapping setup with a Ti:Sapphire laser operating at λ = 790 nm^8,9^ (see Supporting Information, SI). We used a tightly focused circularly polarized spatially phase-modulated beam of light to form an optical ring trap^8,10^. A schematic diagram of the system is shown in Fig. 1a. We trapped a mixture of 150-nm- and 200-nm-diameter Ag NPs and measured their motion via dark-field microscopy at a high frame rate (290fps). The particle positions were tracked^11–13^ and their precisely determined positions were used to calculate the angular position, namely, *θ*_*i*_, of particles *i* = *1*,*2* on the ring. The central angle of the pair, which is denoted as *θ*_*c*_, was defined as the mean angular position of the particles (Fig. 1b). The particle radii were differentiated by their scattering intensity (and image size) on the detector (see SI). We observed directed motion of each electrodynamically interacting pair of dissimilar particles, which is termed a “heterodimer”, toward the larger particle (Fig. 1c and Supplementary videos S2 and S3). By contrast, when two particles of the same size come into close proximity, thereby creating a “homodimer”, they do not exhibit directed motion. These observations are in agreement with forces that we calculated using generalized Mie theory (GMT, see SI), which are shown in Fig. 1d. For a stable optically bound pair^14–16^ (i.e., particles that are separated by ~*λ/n*_*b*_ = *600* nm in water, where *n*_*b*_ is the refractive index) where $\vec F_2 - \vec F_1 = 0$, the transverse force on the pair satisfies $\vec F_{net} = \vec F_2 + \vec F_1 = 0$ only when the two particles have identical radii.^3,4^ The homodimer results can be interpreted as stemming from the conservation of linear momentum due to mirror symmetry between the particles. This symmetry is broken for the heterodimer. While this interpretation would suffice for linearly polarized light, our use of circularly polarized light introduces an equal and opposite (i.e., anti-parallel) force on each nanoparticle that is directed perpendicular to the interparticle separation. These anti-parallel forces create a torque on the dimer and cause it to rotate as a rigid body.^9^ However, full or free rotation was not observed in our experiment because the ring trap is  constricted in its radial direction. The resulting optical gradient force counteracts particle displacements away from the maximal intensity. Manifestations of this torque and its effect will be investigated in future work.

**Fig. 1:**
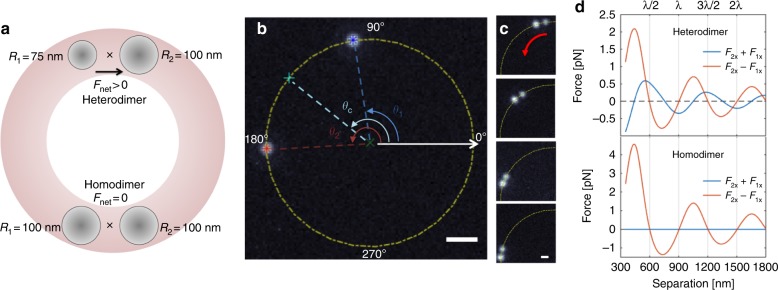
Experimental scheme for measuring "nonreciprocal forces". **a** A schematic diagram of the experiment: Two dissimilar particles in a ring trap (top) experience a net force, namely, $\vec F_{net} \ne 0$, thereby resulting in observable motion. Two identical particles experience $\vec F_{net} = 0$ (bottom). **b** An experimental image and the coordinate system. The trap location is indicated by a dot-dashed yellow circle. The particle locations in the trap are *θ*_*1*_ and *θ*_*2*_. Their mean angular position is *θ*_*c*_. The scale bar is 1 μm. **c** Image sequence of a directed motion event of a heterodimer. When 150-nm and 200-nm-diameter Ag NPs are at optical binding distance, we observe directed motion toward the larger particle. The time difference between the frames is 75 ms and the scale bar is 500 nm. **d** The sum and difference of the forces on both particles (calculated using GMT) as a function of the separation for a heterodimer (top) and a homodimer (bottom). The particle sizes and orientation are identical to those in panel **a**

Figure 2a shows representative time trajectories of *θ*_*c*_ for the homodimer and heterodimers whose images are shown in the insets (see Supplementary videos 1–3; more trajectories are shown in SI). The motion of the pair is directed toward the larger particle and, therefore, can move clockwise or counterclockwise around the ring, depending on the heterodimer orientation. The motion of the heterodimer cannot arise solely from asymmetric hydrodynamic interactions. Hydrodynamic interaction between particles cannot alter the center of the distribution of the Brownian displacements of each of the particles in the heterodimer away from zero displacement without a source of transverse momentum.

**Fig. 2:**
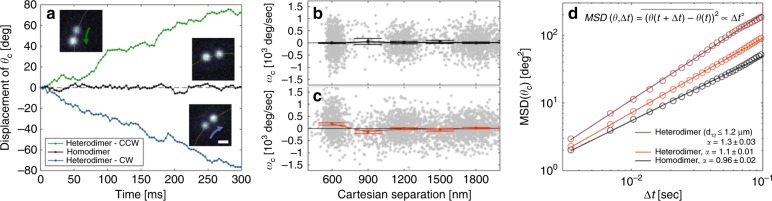
**“**Nonreciprocal” force-induced dynamics. **a** Example trajectories for a homodimer (black) and a heterodimer (color) that are moving in counterclockwise (green) and clockwise (blue) directions. Distribution of instantaneous angular velocities (gray dots) and the mean angular velocities of the homodimers (**b**, black) and heterodimers (**c**, orange) as a function of interparticle separation. The bin size is 300 nm. The mean angular velocity value was calculated by fitting a Gaussian function to the instantaneous velocity distribution. The error bars are the 3σ confidence intervals for fitted means of the distribution. Positive velocity is defined as motion of the heterodimer toward the larger NP. **d** The calculated mean square displacement (MSD) values for the homodimer data that are shown in (**b**) (black), the heterodimer data that are shown in (**c**) (orange), and the subset of the heterodimer data where the interparticle separation was ≤1.2 μm (red). The exponents were obtained from a linear fit of the MSDs shown; individual trajectories are shown in the Supporting Information. The error values are 3σ confidence intervals

We repeated the experiment many times with various nanoparticles and, hence, homodimers and heterodimers (see the Methods section and SI for full details) and combined the results. Figure 2b, c show the angular velocity distributions and the mean angular velocities of the dimer center, which is denoted *ω*_*c*_, as a function of the interparticle separation for the full homodimer and a heterodimer data sets. The instantaneous angular velocity, which is denoted *ω*_*c,n*,_ is defined as the difference in the central angle of the pair in the sequential frames *n,n* + *1* (i.e., $\omega _{c,n} = (\theta _{n + 1} - \theta _n)/{\mathrm{\Delta }}t$, where *n* is the frame number and Δ*t* is the time step). In an overdamped system, $\omega _c \propto \vec F_{net}$. To combine data with different heterodimer orientations, we define positive velocity as the vector from the smaller particle toward the larger particle. Heterodimers exhibit a positive mean angular velocity when the particles are at optical binding separation (600  ± 150 nm) and a negative mean angular velocity when the separation is *3λ/2n*_*b*_ (i.e., 900 ± 150 nm). By contrast, the mean angular velocity for a homodimer is zero for all separations. These observations are in accordance with our prediction from GMT electrodynamics calculations (see Fig. 1d). Both the change in the sign of the mean velocity of particle pairs at optical binding and at *3λ/2n*_*b*_ separations and the motion of the pair toward the larger, thermally hotter particle, demonstrate that the driven motion is a result of the electromagnetic field and not heating-induced self-thermophoresis^17^ (see SI for details).

Figure 2d shows the (average) mean square displacement (MSD) of *θ*_*c*_ for the homo and heterodimer trajectories. The exponent, α, of $MSD\left( {{\mathrm{\Delta }}t} \right) = D \cdot {\mathrm{\Delta }}t^\alpha$ (with diffusion coefficient D and lag time ∆t) for the homodimer is α = 0.96 ± 0.02, as expected for a diffusing Brownian particle.^18^ For heterodimers, we observe *α* > 1, which indicates driven motion^19^, and an even greater value, namely, α = 1.3 ± 0.03, when we only consider trajectories for which the particle separation is less than 1.2 μm; that is, two optical binding separations. This value was chosen to allow longer trajectories for analysis (see SI for more details about the number of experiments and the trajectories that were analyzed).

Our findings are related to recent publications that report the calculation and measurement of the dynamics that result from an asymmetry in the linear or angular momentum of the light that is scattered by optically trapped objects^20,21^ in a tractor beam configuration. We extended previous theoretical work, which considered particles in a linearly polarized beam,^3^ to circular polarization to explain the self-motility of electromagnetically interacting dimers (see SI for a detailed discussion). We also simulated the dynamics of Ag NP dimers using GMT.^22,23^ Each dimer, which consisted of two spherical Ag NPs with radii *R*_*1*_ and *R*_*2*_ that were separated by a distance *d* along the *x*-axis, was placed in a water medium (*n*_*b*_ = 1.33) with an incident right-handed-circularly (RHC) polarized plane wave (of 800 nm vacuum wavelength). Forces were calculated by integrating the Maxwell stress tensor over a closed surface surrounding the particles. This calculation enforces conservation of linear momentum. Simulations were performed in which *R*_*2*_ was varied for three values of *R*_*1*_ at a separation of *d* = 600 nm (Fig. 3a). When *R*_1_ = *R*_2_ $\vec F_{net,x} = 0$ vanishes, as expected for the homodimer. When *R*_1_ < *R*_2_ $\vec F_{net,x} > 0$ causing the heterodimer to move in the +*x*-direction. If *R*_1_ > *R*_2_ the net force is reversed and the heterodimer moves in the *–x*-direction. In both cases the motion is in the direction from the small particle to the larger one.

**Fig. 3:**
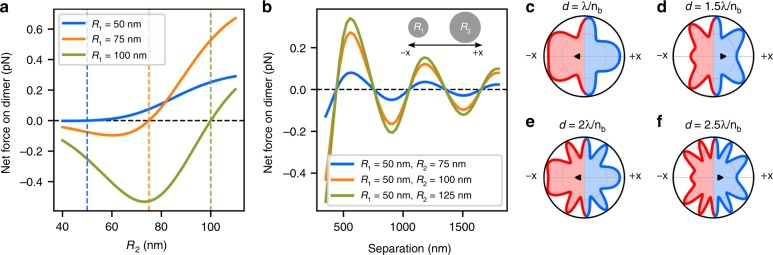
Simulations of heterodimers that use the GMT method for the calculation of forces on electrodynamically interacting dimers. **a** The net force on the dimer, *F*_*net,x*_, as a function of the radius of particle 2 with three different radius values for particle 1: 50 nm, 75 nm, and 100 nm. The dashed lines indicate the cases of the three homodimers, where *F*_*net,x*_ vanishes. **b** *F*_*net,x*_ vs. separation for three heterodimers. **c**–**f** Angular scattering intensity in the *xy*-plane from the *R*_*1*_ = 75 nm and *R*_2_ = 100 nm heterodimer for various dimer separations *d*. The black triangle indicates the center of mass (“CM”) of the angular distribution. We define the positive *x*-direction to be pointing from the smaller particle to the larger particle. Stable optical binding configurations (*d* = *λ, 2λ*) scatter more in the negative *x*-direction, whereas unstable configurations (*d* = *1.5λ* and *2.5λ*) scatter more in the positive *x*-direction

Additional simulations were performed for fixed nanoparticle radii with varying separation from *d* = *λ/2n*_*b*_ to *d* = *3λ/n*_*b*_. Figure 3b shows the net force on the heterodimers as a function of *d*: $\vec F_{net,x} > 0$ at separations near 600 nm and 1200 nm, i.e., at stable optical binding configurations; and $\vec F_{net,x} < 0$ for particle separations near 900 nm and 1500 nm, where the heterodimer is also in an unstable configuration. Increasing the size of the larger nanoparticle increases *F*_*net,x*_, but does not otherwise change the functional form of the force curves.

For our total system (particle and fields) to conserve linear momentum, the total momentum that is carried by the electromagnetic field that is scattered from the particle pair must be equal and opposite to the induced momentum of the dimer. Figure 3c–f shows a separation-dependent imbalance of angular scattering due to dipolar interference, i.e., more light is scattered in one direction than in the other. For *d* = *λ/n*_*b*_ and *2λ/n*_*b*_ (stable optical binding configurations), more light is scattered in the *–x-*direction and the net force that acts on the dimer is in the +*x-*direction. Similarly, for *d* = *3λ/2n*_*b*_ and *5λ/2n*_*b*_ (unstable configurations; see Fig. 1d), more light is scattered in the + *x-*direction, which corresponds to a net force in the *–x*-direction. This asymmetry in the far-field angular scattering creates a force on the dimer, thereby setting it in motion. The simulation results also confirm the switching of sign of the force observed in our experiments (Fig. 2b) for various particle separations. Note that asymmetric scattering has been reported for plasmonic Yagi–Uda nanoantennas that were fabricated on a fixed substrate.^24,25^

Since the electrodynamically interacting NP pairs can be treated as a single (a)symmetric scatterer, a similar reactive optical matter effect, namely a “photophoretic” drift force,^26^ is expected for particles (in a ring trap) that have asymmetric shapes and exhibit asymmetric scattering^27^. We used the same experimental approach to study asymmetric NPs and aggregates; specifically, touching gold nanostar dimers and a large asymmetric aggregate of gold nanoparticles shown in Fig 4a. The latter also interacts with many single Au NPs in a ring trap. We use linearly polarized light instead of circularly polarized light to avoid causing the asymmetrical “particles” to rotate (spin).^28,29^

As shown by the time-trajectory in Fig. 4c, the nanostar dimer oscillates between a position parallel (*θ*_*c*_ *≈* 270°*)* and perpendicular (*θ*_*c*_ *≈*180°*)* to the light polarization. It drifts with a direction tangential to the ring and changes orientation at the 180° and 270° extremes of its range of motion. The restricted range of motion and switching of orientation results from its interaction with the polarized light and from occasional interactions with neighboring nanostars (Supplementary video S4). The several large variations of the velocity and the resultant MSD with α ≈1.39 ± 0.01 that is calculated from the time-trajectory confirm the directed (driven) motion: the nanostar dimer is strongly driven in the parts of the trajectory that transition between the 180° and 270° limits. See SI for further elaboration. The unknowable shapes of the nanostar particles preclude correspondingly accurate simulations. Further study of the motion control is the subject of ongoing research.

**Fig. 4:**
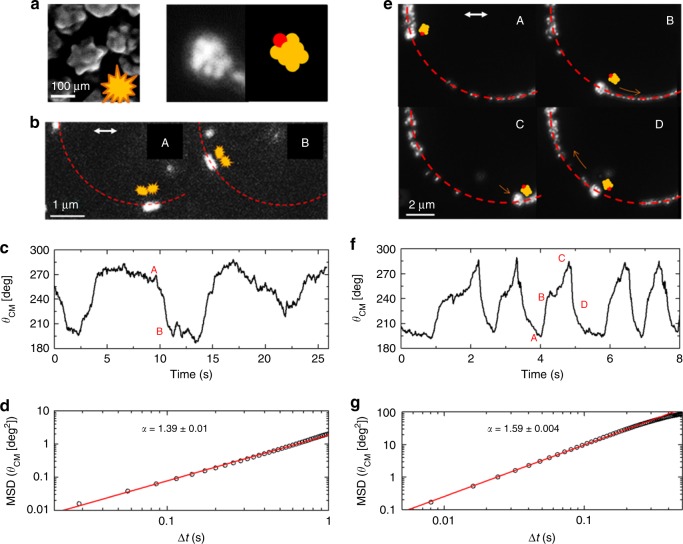
Demonstration of asymmetric forces on asymmetric nanoparticle structures. **a** An SEM image of nanostars and a dark-field optical image of the Au–NP aggregate. Schematic diagrams (avatars) highlight their asymmetric structures and orientations. The red point defines the orientation. Dark-field images of (**b**) the nanostar dimer and (**e**) the Au–NP aggregate in the ring trap (highlighted in red). The white arrow indicates the polarization direction of the trapping beam. **c** and **f** show trajectories of the two asymmetrical objects. Both asymmetrical objects exhibit highly oscillatory velocity. The notable differences in their dynamics are discussed in the Supplementary Information. **d** and **g** are the MSDs that are calculated from the trajectories. The lower frame rate (35 fps for the nanostar and 82 fps for the aggregate) is still adequate for capturing the highly driven nature of the dynamics: α = 1.39 for the nanostars and α = 1.59 for the Au–NP aggregate, including the several velocity reversals

Similar results are obtained for the Au–NP aggregate. Figure 4e shows that it oscillates between ≈180° and ≈270°. It exhibits driven motion with the orientations that are shown by the avatars in Fig. 4a,e (reversing orientation at the two extreme positions; see SI). MSD analysis demonstrates strong driven behavior with α = 1.59. See SI for further details and discussion.

In this letter, we have experimentally demonstrated driven motion of both Ag NP heterodimers and intrinsically asymmetric scatterers in optical ring traps, namely, 1-D plane wave fields. Our electrodynamic simulations indicate that the net force on a dimer is accompanied by a net asymmetric scattering in the opposite direction. Therefore, we attribute the driven (reactive) motion of asymmetric optical matter systems to the conservation of linear momentum. Fundamentally, this self-motility follows from Noether’s theorem and the conservation of total momentum of particles and fields for systems with broken mirror symmetry^30^. While these experiments were conducted with nanoparticles and aggregates confined to a ring trap, the results are applicable to any optically trapped matter structure that exhibits an electromagnetic asymmetry.

Generating directed motion at the nanoscale is challenging^31^ due to the overdamped nature of dynamics at low Reynolds number and the Brownian forces that are antithetical to orientational control of nanoscale objects. Optical trapping offers a variety of solutions to these challenges since it enables precise control over the positions and orientations of trapped particles. Although systematic driving forces can be applied via the use of phase gradients, apparent nonreciprocal forces, such as those that are explored above, create self-motile particles that do not require specific chemical environments or chemical fuels^32^ or complex structures.^33^ Therefore, optically controlled asymmetric nanoparticle assemblies, such as those that are reported here, can be used as active colloids^32^ and fully controllable “nanoswimmers” for research in soft condensed matter and biophysics.

## Methods

### Optical trap details

We used a CW Ti:Saphire laser operating at 790 nm (vacuum wavelength) to form a ring trap with a radius of 3.4 μm. The trap diameter was chosen to minimize the effect of scattering forces from particles that might be present in other sections of the ring. The laser beam was focused into a sample cell that contained a mixture of 150-nm-diameter and 200-nm-diameter Ag nanoparticles that were coated with a ligand layer of polyvinylpyrrolidone (PVP; NanoComposix) whose concentration was diluted from the stock solution with 18MΩ deionized water at a ratio of 1:200.

### Particle imaging and tracking

Following data acquisition, we tracked the particle positions using the Mosaic particle tracking toolbox for ImageJ^11^. Due to the small size of the particles on the detector, we applied the localization algorithm with a small fitting window. This introduced pixel locking error, in which the particle positions were localized toward the center of the pixels. The pixel locking error was corrected (removed) by applying the single pixel interior fill factor (SPIFF) algorithm^12,13^.

### Particle characterization

The 150-nm- and 200-nm-diameter Ag nanoparticles were differentiated by imaging them on the sCMOS array detector (Andor, Neo) and observing differences in their relative size and brightness. The 200-nm-diameter particles appeared larger on the sCMOS (i.e., occupied more pixels on the detector) and brighter compared to the 150-nm-diameter particles. We coupled the dark-field scattered light out through the side port of the inverted microscope and into a spectrometer (Shamrock-Andor SR 193i-BI-SIL) to estimate the individual particle sizes by measuring the spectral responses of individual particles and comparing them to Mie theory scattering calculations; full details are given in the Supporting Information. We state that we used 150nm diameter and 200nm diameter Ag NPs for the experiments based on the manufacturer's stated specifications (Nanocompsix). However, as shown in the Supplementary Information, we determined that the actual typical diameters of the larger nanoparticles is 175-185nm.

### Data analysis

We performed 11 independent experiments, each of which was 7000 frames in length. Of these experiments, we limited the analysis to cases in which we observed two particles in the trap without a third particle nearby. We used the intensity information from the sCMOS detector to identify whether the particle pair was a homodimer (five particle pairs, 8500 frames) or a heterodimer (12 particle pairs, 18,900 frames). These combined data enabled us to bin the mean angular velocity of the dimers, namely, *ω*_*c*_, as a function of interparticle separation, as shown in Fig. 2b, c. We calculated the MSD and the transport exponent, *α*. Separation-dependent MSD curves were calculated by identifying 9 trajectories of homodimer pairs and 11 trajectories of heterodimer pairs that were at optical binding separation (less than 1.2 μm). Then, we used their trajectories to calculate the red MSD curve that is shown in Fig. 2d; full details and time trajectories of the homodimers and heterodimers are given in the Supplementary Information.

## Electronic supplementary material

Supplemental material pdf

Video 1 - Ag homodimer in ring trap

Video 2 - Ag heterodimer in ring trap - CW motion

Video 3 - Ag heterodimer in ring trap - CW motion

Video 4 - nanostar dimer in ring trap

Video 5 - Au cluster in ring trap
