# Supplementary material for: Reactive optical matter: light-induced motility in electrodynamically asymmetric nanoscale scatterers
Source: Light Sci Appl. 2018 Dec 12;7:105. doi: 10.1038/s41377-018-0105-y (PMC6289991; doi:10.1038/s41377-018-0105-y)
Supplement: Supplementary file 1 — Supplemental material pdf [file 41377_2018_105_MOESM1_ESM.pdf]

Supplemental information for:
Reactive optical matter: light-induced motility in electrodynamically asymmetric nanoscale scatterers

Yuval Yifat,[1,†] Delphine Coursault,[1,†] Curtis W. Peterson, [1,2,†] John Parker, [1,3,†] Ying Bao, [1,4] Stephen K. Gray, [5] Stuart A. Rice, [1,2] and Norbert F. Scherer[*,1,†]

1. James Franck Institute, The University of Chicago, 929 E. 57th Street, Chicago, Illinois 60637, USA

2. Department of Chemistry, The University of Chicago, 929 East 57th Street, Chicago, Illinois 60637, USA

3. Department of Physics, The University of Chicago, 929 East 57th Street, Chicago, Illinois 60637, USA

4. Present address: Department of Chemistry, Western Washington University, 516 High Street, Bellingham, WA 98225, USA

5. Center for Nanoscale Materials, Argonne National Laboratory, 9700 South Cass Avenue, Argonne, Illinois 60439, USA

†These authors contributed equally

*Corresponding Author, email: nfschere@uchicago.edu

Experimental setup

A diagram of the setup used to trap the 150nm and 200nm Ag nanoparticles is shown in Figure S1(a). The setup consisted of a continuous wave Ti:sapphire laser emitting linearly polarized light at a wavelength of 790 nm. The beam was collimated and reflected off a spatial light modulator (SLM; BNS/Meadowlark HSPDM512-785nm), which was used to shape the beam by imparting the phase necessary for a ring trap. The phase mask used in the experiment is shown in Figure S1(b). The beam was reflected from a dichroic mirror and into an inverted optical microscope (Nikon Ti), through a quarter wave plate, which is used to control its polarization (*i.e.* convert from linear to circular), and through a 60x IR corrected water immersion objective (Nikon 60x Plan APO IR water immersion objective, NA=1.27). The total optical power of the trapping laser measured before the dichroic mirror was 150 mW, giving a power density of 1.5 MW cm^{-2} at the focus.

Figure S1(c) is an image of the ring trap. In order to measure the beam dimensions we removed the near-IR filter before the sCMOS detector (Andor Neo; 6.5 μm pixel size) and imaged the reflection of the beam off the coverslip. The beam was focused slightly beneath the top coverslip. The ring was measured to have a radius of 3.4 μm and a 500 nm width (i.e. FWHM) of the annulus. In addition to the ring trap that was used in the experiments, there was a noticeable focused Gaussian beam in the center of the ring trap (i.e. a zero-order reflection from the SLM) that had no effect on the experimental results due to its large distance (R=3.4 μm) from the particle locations on the ring.

The beam was focused into a sample cell that was filled with a solution of 150 nm and 200 nm silver nanoparticles coated with polyvinylpyrrolidone (PVP). The stock solutions were diluted in 18 MΩ DI water at a ratio of 1:200. The particles were illuminated using a dark-field condenser, and the light they scattered was captured by the objective and imaged onto a sCMOS detector with a total magnification of 90x, giving an effective pixel size of 72 nm. The particle motion was captured in a 120x120 pixel region of interest on the detector with an exposure time of 1 ms at a frame rate of 289 frames per second.

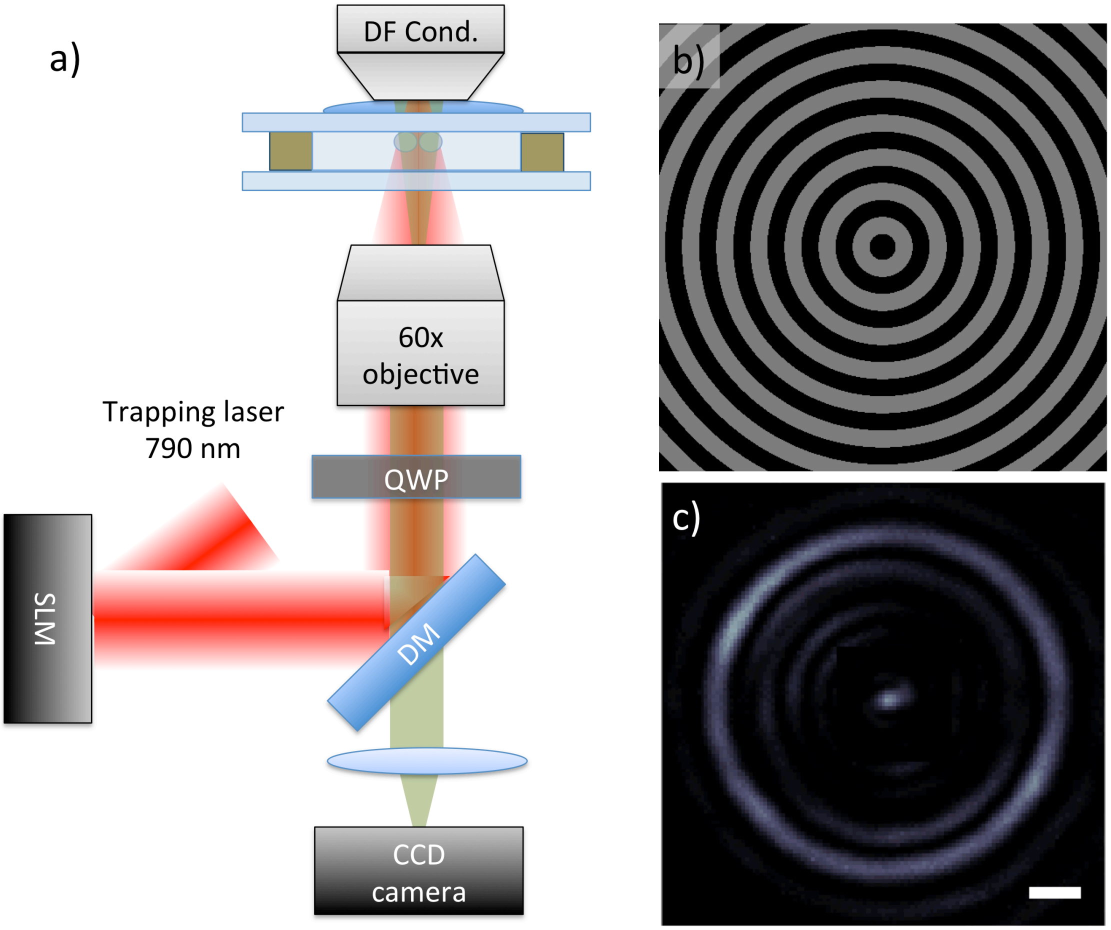

Figure S1: (a) Diagram of experimental trapping setup described in the text. SLM-Spatial Light Modulator, DF Cond. - Dark-field condenser, DM - Dichroic mirror. QWP Quarter wave plate. Trapping laser is reflected from the SLM which is used for beam shaping. Dark-field (high N.A.) illumination that scatters from the Ag nanoparticles is collected by the microscope objective, spectrally filtered and imaged to a sCMOS array detector. (b) The phase mask used to create the ring trap used in our experiments. The phase mask only uses two phase levels (black pixel level = 0 phase shift, gray pixel level = π phase shift). (c) Image of the ring trap on the sCMOS detector. The Gaussian spot in the center is the zero-order reflection of the trapping laser from the SLM. The spot did not affect our experiments due to the large diameter of the trap. Scale bar is 1μm.

Characteristics of Ag nanoparticles

The particles used for the trapping experiments described in the main text are an equal mixture of 150 nm diameter and 200 nm diameter PVP coated silver nanoparticles (NanoComposix; 150 nm diameter: NanoXact Silver KJW1882 0.02 mg ml^{-1}; 200 nm diameter: NanoXact Silver DAC1326 0.02 mg ml^{-1}). Each stock solution was diluted in DI water (at a ratio of 1:200) and equal volumes were combined.

The identification of the different sized particles was achieved by analyzing their relative brightness and size on the sCMOS detector. See Figure S2(a,c) for representative images of a 150 nm (Fig S2(a)) and a 200 nm (Fig S2(c)) Ag nanoparticle imaged with our optical setup. The visual difference between the two particle images was verified as being due to their physical size by a spectroscopic measurement. Individual particles were captured in a Gaussian trap and the light scattered from them was directed through the side port of the microscope, into a spectrometer (Andor Shamrock 193 imaging spectrograph; SR 193i-B1-SIL), and detected with an EM-CD array detector (Andor Newton).

Figure S2(b,d) shows the spectral measurement for the particles imaged in Figure S2(a,c) along with the expected scattering cross-section calculated from Mie theory[S1]. As can be seen in Figure S2(b), the spectral measurement from a trapped 150 nm diameter Ag nanoparticle is in agreement with the calculated Mie scattering spectrum. The abrupt decrease in signal at 750 nm is due to a near-IR filter placed after the dichroic mirror to block the reflected laser light. On the other hand, Figure S2(d) shows that the the spectral response of the 200 nm Ag nanoparticle is blue-shifted compared to the expected theoretical scattering for a 200 nm particle, and is in closer agreement with the spectrum calculated for a 175 nm Ag nanoparticle. Repeating this experiment for different particles showed a variance in the measured spectral response from the 200 nm particles, and a consistent spectral result from the 150 nm particles.

The plethora of spectra for 200 nm diameter Ag nanoparticles implies dispersion in size or shape or both. This size (shape) dispersion was confirmed by electron microscopy imaging of

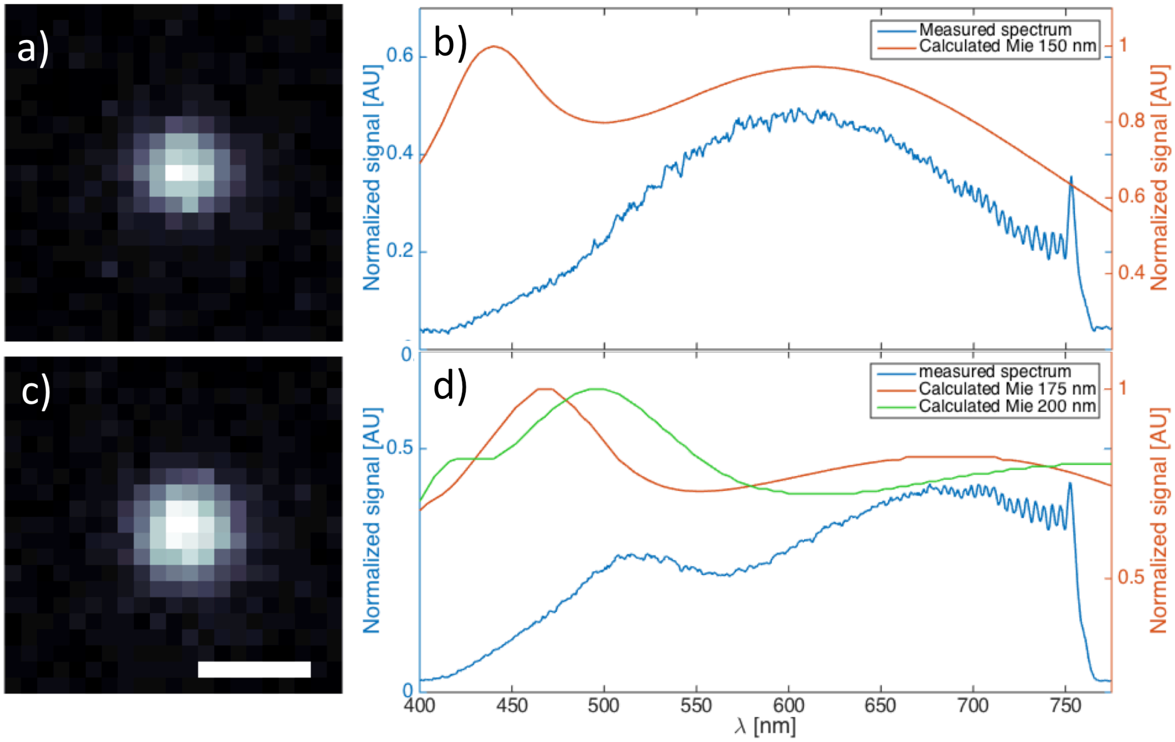

Figure S2: (a) Representative image of a 150 nm diameter Ag nanoparticle trapped in a Gaussian beam. (b) Measured scattering spectrum (blue) from the 150 nm diameter particle shown in panel (a) as well as the calculated theoretical Mie scattering for 150 nm diameter Ag nanoparticle suspended in water (red). Spectra were measured by directing the scattered light through the side port of the inverted optical microscope to a spectrometer. The abrupt drop in signal from 750 nm is due to a near-IR notch filter used to block scattered light from and reflections of the trapping beam reaching the detector. Conversely, light from 500 - 750 nm was used to image the nanoparticles by dark-field microscopy. (c) Representative image of a 200 nm diameter Ag nanoparticle trapped in a Gaussian beam. Intensity scales of images (a) and (c) are identical. Scale bar is 500 nm and applies to (a) and (c). (d) Measured scattering (blue) from the 200 nm diameter particle shown in panel (c) as well as calculated theoretical Mie scattering for 175 nm (red) and 200 nm (green) diameter Ag nanoparticles suspended in water.

the different sized nanoparticles. The particles were drop cast on a copper grid and imaged using a Transmission Electron Microscope (TEM; FEI Tecnai F30 300kV FEG) using a magnification of 145,000x (see Fig S3(a,c)). The 150 nm Ag particles are uniform in size and nearly spherical in shape (but with facets), whereas the 200 nm particles were noticeably less spherical and less uniform in size.

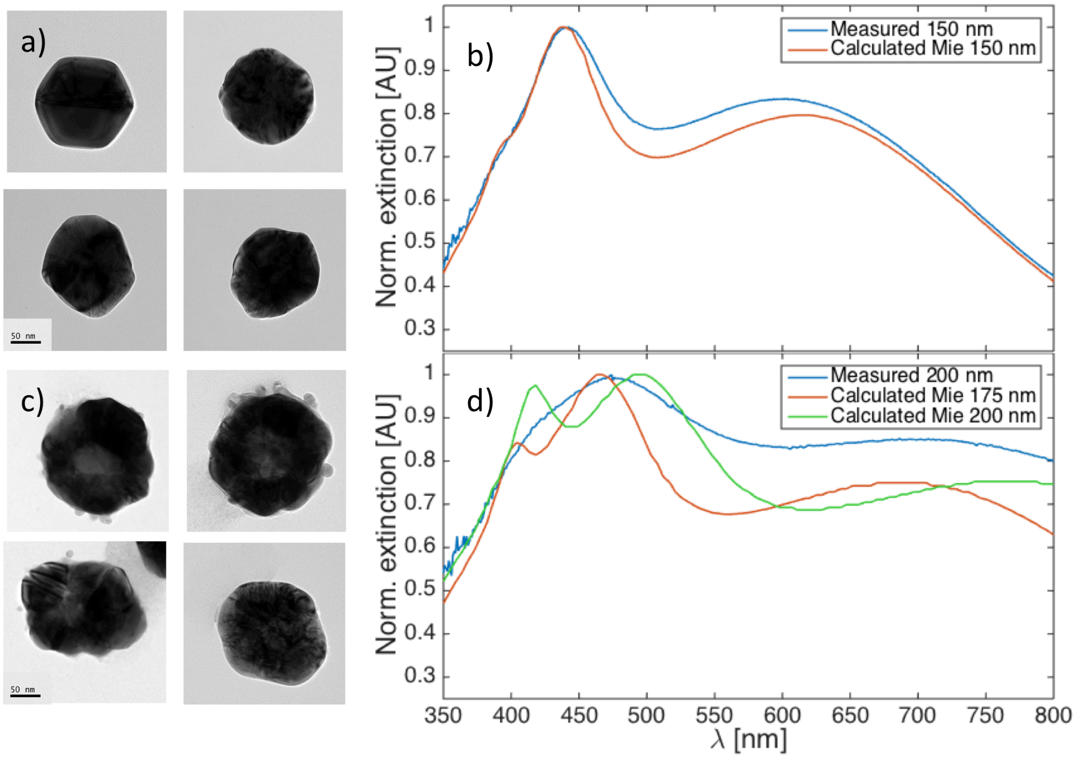

Figure S3: Transmission electron microscopy (TEM) and spectral analysis of Ag nanoparticles. (a) TEM images of 150 nm diameter Ag nanoparticles dispersed on a copper grid. Scale bar is 50 nm. (b) Normalized extinction spectrum of 150nm diameter Ag nanoparticle stock solution taken with a UV-Vis-IR spectrophotometer (blue curve). Peak maxima at 440 nm and 600 nm correspond to the dipole and quadrupole Mie scattering modes of a 150 nm diameter silver particle immersed in water (calculated values given as the red curve). (c) TEM images of 200 nm diameter Ag nanoparticles dispersed on a copper grid. Note the larger variance in size and shape. Scale bar is 50 nm. (d) Normalized extinction spectrum of 200nm diameter Ag nanoparticle stock solution taken with a UV-Vis-IR spectrophotometer (blue curve). Peak maxima are at 475 nm and 700 nm. Green and red curves respectively correspond to calculated Mie extinction spectra of a 175 nm and 200 nm diameter silver particle immersed in water. Note the broad width of measured peak compared to that of the the calculated values and the measured value from panel (b), implying a dispersion in particle diameters (and shapes) in the stock solution.

We also measured the ensemble extinction of the two stock solutions using a Cary 5000 UV/Vis/IR spectrophotometer (see Fig S3(b,d)). For the 150 nm diameter Ag nanoparticle solution we observed peaks at 440 nm and at 600 nm (blue curve in Fig S3(b). As there is good agreement between the measured and calculated resonance locations, these extinction peaks are assigned as the dipole and quadrupole modes calculated from Mie extinction of a silver particle of the same diameter immersed in water (red curve). However, for the 200 nm diameter Ag nanoparticle stock solution, we observed much broader extinction peaks at 475 nm and 700 nm (blue curve in Figure S3(d)). These peaks are wider than expected for a monodisperse suspension of Ag nano particles with a diameter of 175 or 200 nm (compare to red and green curves in Figure S3(d)). This implies that the solution is not monodisperse and is an ensemble of many different particle diameters with a mean value of around 180 nm. The reason for this non-uniformity results from the well-established difficulty in synthesis of Ag nanoparticles larger than 150 nm[S2].

Despite their non-uniformity, the "200 nm" Ag particles are consistently larger than their 150 nm counterparts and this size difference manifests itself in the non-reciprocal dynamics shown in the main text.

Theoretical description of non-reciprocal forces

An expression for the net optical force on a dimer (of spherical isotropic particles A and B) in a plane transverse to the propagation of plane-wave illumination can be obtained in the point dipole approximation. The component of the electric field in the i direction at particles A and B (at only the first order of scattering)[S3] is

$$E_A^i = E_0^i + G_{ij}^{AB} E_0^j \alpha^B; \quad E_B^i = E_0^i + G_{ij}^{BA} E_0^j \alpha^A \tag{S1}$$

where E_0^i is the incident electric field, α^A or α^B is the polarizability of particle A or B, and G_{ij}^{AB} are the elements of the dyadic Green's function for the vector between particles A and

B. If we assume that the particles lie on the x axis, only the diagonal elements of G_{ij}^{AB} are non-zero. For a circularly polarized plane wave propagating in the z direction this leads to a net force in the x direction F_x^{net} on the dimer

$$F_x^{net} = \frac{E_0^2}{2}\mathrm{Re}\left[(\alpha^{A*}\alpha^B - \alpha^A\alpha^{B*})\frac{\partial}{\partial x}(G_{xx}+G_{yy}) + (\alpha^{A*}|\alpha^B|^2 - |\alpha^A|^2\alpha^{B*})\left(\frac{\partial G_{xx}}{\partial x}G_{xx}^* + \frac{\partial G_{yy}}{\partial x}G_{yy}^*\right)\right]. \tag{S2}$$

This equation extends the treatment derived in *Sukhov et. al.*[S4] from particles trapped in a linearly polarized plane wave to a plane wave with circular polarization.

The corresponding result for a pair of particles (point dipoles) interacting in a beam linearly polarized along the x-axis (inter-particle axis) is

$$F_x^{net} = \frac{E_0^2}{2}\mathrm{Re}\left[(\alpha^{A*}\alpha^B - \alpha^A\alpha^{B*})\frac{\partial G_{xx}}{\partial x} + (\alpha^{A*}|\alpha^B|^2 - |\alpha^A|^2\alpha^{B*})\left(\frac{\partial G_{xx}}{\partial x}G_{xx}^*\right)\right], \tag{S3}$$

which after rearrangement is identical to the result in *Sukhov et al*[S4] except for a factor accounting for infinite-order interactions between the two particles. The additional factors of $\frac{\partial G_{yy}}{\partial x}$ and $\frac{\partial G_{yy}}{\partial x}G_{yy}^*$ in equation S2 for the case of circular polarization affect the dependence of the derived forces on interparticle separation. However, equations S2 and S3 are qualitatively similar. Both equations equal zero when $\alpha^A = \alpha^B$, in accordance with the experimental and simulation results presented in the main text. In fact, both expressions vanish if the two polarizabilities are proportional by a factor of a real number (e.g. $\alpha^A = C\alpha^B$ where C is a real number). *Therefore, it is necessary that the α^A and α^B have different angles in the complex plane for the non-reciprocal forces to exist within this approximation.* In summary, our analytical results show that non-reciprocal forces arise in pairs of particles with dissimilar polarizabilities under both linear and circular polarization, although the exact spatial dependence of these forces is different for those two cases.

Analysis of combined particle trajectories

We performed 11 independent experiments, each of which was 7,000 frames in length. Of these experiments we limited our analysis to frames in which we observed two particles in the trap without another particle nearby. We then used the intensity information from the sCMOS detector to identify whether the particle pair was a homodimer (5 experimental videos, 8,500 total frames) or a heterodimer (12 experimental videos, 18,900 total frames).

From each video frame we localized the particle centroids using particle-tracking algorithms (*e.g.* Mosaic in ImageJ[S5])and used their positions to calculate the interparticle separation θ_c. The motion of their mean angular position (or "center of geometry") ω_c was calculated by how much their mean angle changed between consecutive frames, *i.e.* for frame n, we define $\omega_{c,n} = \frac{\Delta\theta_n}{\Delta t} = \frac{\theta_{c,n+1}-\theta_{c,n}}{\Delta t}$ where $\theta_{c,n}$ is the mean angular position of the two particles in frame n, and Δt is the time step. This data was binned by interparticle separation, d_{12}, and used to produce the plots of ω_c as a function of d_{12} in Fig 2(b,c). By plotting the motion of the central interparticle angle as a function of interparticle separation we found the mean rotational velocity of a homodimer (Fig 2b in the main text) and a heterodimer (Fig. 2c in the main text). Fig S4 shows the distribution of ω_c along with the Gaussian fit for the homodimers and the heterodimers. The FWHM of the Gaussian fits are due to the thermal Brownian fluctuations inherent in the experiment. It is important to note that the error bars shown in Figure 2(b,c) in the main text are the 3σ confidence interval for the center of the Gaussian fits, and thus, despite the width of the Gaussian distribution, its central ω_c value is statistically significant.

The MSD results and the fitted transport exponents, α, for the entire homodimer and heterodimer dataset (*i.e.* $MSD(\theta_c|\forall d_{12})$, where d_{12} is the interparticle separation) were calculated by aggregating the trajectories from the entirety of the experimental videos identified above (*i.e.* all 8,500 homodimer video frames or 18,900 heterodimer video frames). These MSD curves are shown in the main text as the black and orange curves in Fig. 2(d).

Calculation of the MSD for cases where the particles were optically bound (*i.e.* $MSD(\theta_c|d_{12} <$

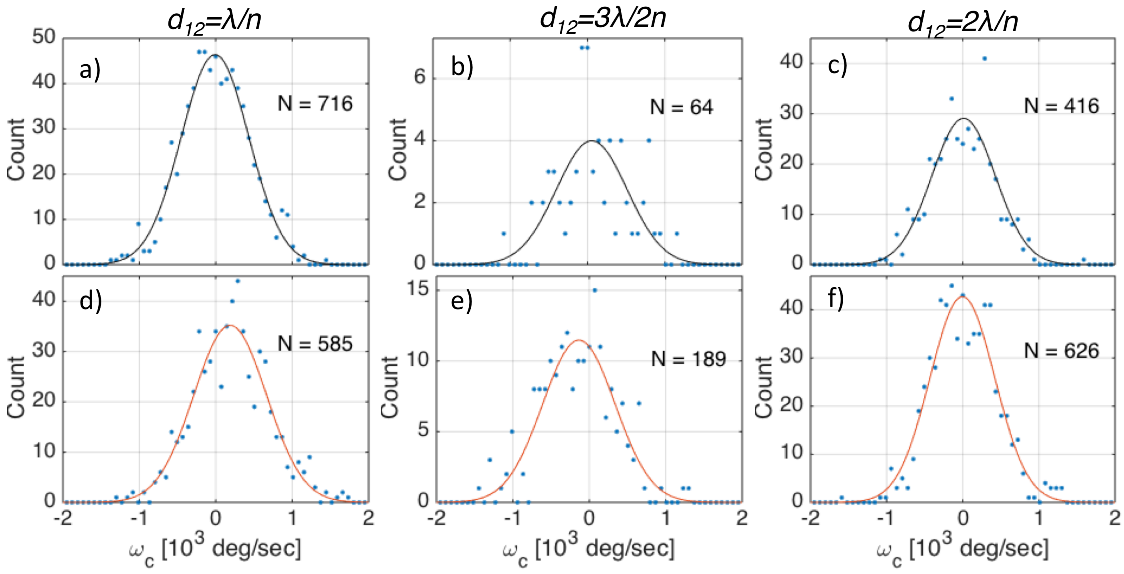

Figure S4: Distributions and Gaussian fits to the homodimer (a-c) and heterodimer (d-f) dimer velocity, ω_c, data shown in the main text in Figure 2(c,d). Different columns represent particles separated by one optical binding distance (a,d), 1.5 optical binding distance (b,e) and two optical binding distances (c,f). (a) Histogram of instantaneous angular velocity, ω_c, for homodimers where the particles are at one optical binding separation ($450 \leq d_{12} < 750$ nm). Center of the Gaussian curve is at -9 ± 26 deg s^{-1} (mean ± S.D). (b) Homodimer velocity data for the first unstable separation (($750 \leq d_{12} < 1050$ nm). Center of Gaussian fit is at 54 ± 130 deg s^{-1}. (c) Homodimer velocity data for the second optical binding separation (($1050 \leq d_{12} < 1350$ nm). Gaussian center is at 11 ± 52 deg s^{-1}. (d) Histogram of instantaneous angular velocity, ω_c, for heterodimers where the particles are at one optical binding separation ($450 \leq d_{12} < 750$ nm). Center of the Gaussian fit (orange curve) is at 190 ± 50 deg s^{-1}. (e) Heterodimers velocity data for the first unstable separation (($750 \leq d_{12} < 1050$ nm). Center of Gaussian fit is at -136 ± 70 deg s^{-1}. (f) Heterodimers velocity data for the second optical binding separation (($1050 \leq d_{12} < 1350$ nm). Center of Gaussian fit is at 5 ± 40 deg s^{-1}. The values of N in each panel indicate the total counts (events) in each histogram.

1.2 µm)) was done by selecting and analyzing the portions of the experimental trajectories where the interparticle separation was small enough for the particles to interact electrodynamically. Fig S5 shows the trajectories of 9 bound homodimers (a) and 12 bound heterodimers (b) from our experimental set. The start time of the trajectories was selected as when the interparticle separation was less than two optical binding separations (1.2 µm), and ended when the interparticle separation was greater than 1.5 µm for more than one time step. These values were chosen to allow analysis of cases in which the particles fluctuated away from optical binding separation for short periods of time. Fig S5(c,d) shows the calculated MSD values of the trajectories shown in Fig S5(a,b), as well as the mean MSD (connected grey diamonds) obtained by aggregating over the all the bound homodimer or heterodimer trajectories. The aggregated MSD curve for the bound heterodimer is identical to the orange curve shown in Figure 2(d) the main text. The Mean homodimer MSD shown in Fig S5(c) has a slightly different exponent than that of the entire homodimer population regardless of separation (black curve in Fig 2(d) in the main text). The reason for the slight difference between the exponent values ($\alpha = 0.96$ vs. $\alpha = 1.0$) is that the MSD shown in Fig S5(c) was fitted only to the trajectories in which the particles are at optical binding (*i.e.* $d_{12} \leq 1.2$ μm). Conversely, the exponent shown in Fig 2(d) in the main text was obtained by fitting the *entire* trajectory information. Trajectories that are shorter than 35 time steps (roughly 0.1s) are not shown in Figure S5. The value of α was unchanged whether or not the trajectories shorter than 35 time steps were used to calculate the MSD in addition to the longer trajectories.

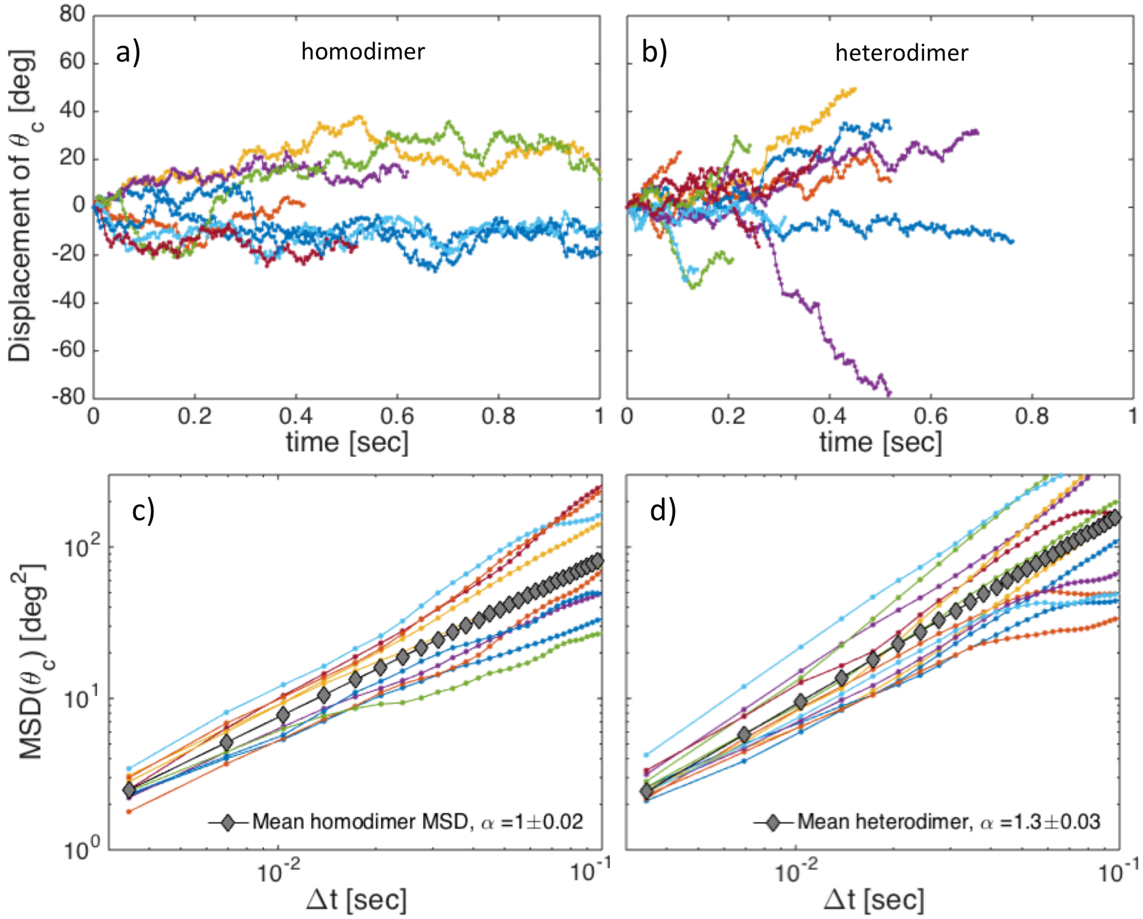

Figure S5: Experimental trajectories and MSD calculations for bound homodimers and heterodimers. (a-b) Time series of angular displacement for homodimers (a) and heterodimers (b). Trajectories were started when the particles were separated by less than 1.2 µm, and ended when the particles were separated by more than 1.5 µm for longer than one time step. This was done to include trajectories in which the particles fluctuate out of optical binding for short periods of time and to allow aggregation of long trajectories. Particle size was determined from the scattering intensity recorded by the sCMOS detector. (c-d) MSD values of trajectories for homodimer (c) and heterodimer (d). The different colors correspond to the trajectories shown in panels (a,b). The mean MSD value (marked as gray connected diamonds) is the mean MSD calculated from all the individual trajectories shown in (a,b). The mean heterodimer MSD is identical to that shown in the Figure 2(d) in the main text.

The MSD calculated from a single heterodimer trajectory demonstrates the driven nature of the heterodimer motion[S6]. Figure S6 shows an example of the MSD calculated from a single heterodimer trajectory (specifically the trajectory of the heterodimer driven in the CW direction motion shown in Fig 2(a) in the main text), along with a quadratic fit that signifies driven motion.

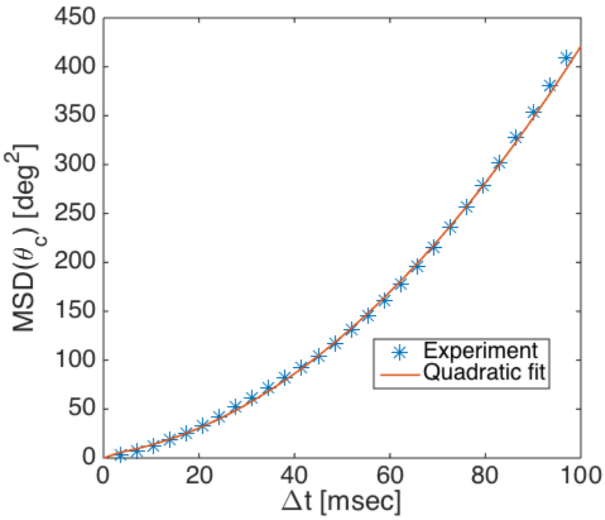

Figure S6: MSD calculated from a single heterodimer trajectory, specifically the CCW heterodimer trajectory shown in Fig 2(a) in the main text. The MSD was fitted with a quadratic function, demonstrating the driven nature of the motion.

Effect of nanoparticle heating

It is well established that micro- and nano-scale Janus particles exhibit driven motion through self-thermophoresis.[S7,S8] A Janus particle is usually designed so that half of its surface area is coated with a material such as Au which absorbs the laser light. When such a particle is illuminated it will heat the environment around the coated area and exhibit driven motion towards its cooler, uncoated side due to increased thermal fluctuations on the heated side. The driven motion we observe in this manuscript is significantly different from self-thermophoretic motion. In this section we will describe the observed differences between self thermophoresis and electrodynamically driven motion.

The first important point: if the cause of the motion of the heterodimer were solely due to self-thermophoresis, one would expect that increasing the interparticle separation would cause a monotonic decrease in the driven component of the pair. Essentially, as the inter-particle separation increases, they become less like a Janus particle and more like 2 independent particles so the driven component in their motion should decrease. However, as we show in Fig. 2c in the main text, when the particles are separated by $3\lambda/2$ we observe a statistically significant motion in the *opposite* direction (*i.e.* towards the smaller particle). This change in the direction of the directed motion cannot be explained by self-thermophoresis, and supports our observation that the motion is due to oscillatory electromagnetic interaction.

Another distinction from self-thermophoresis is the direction of the directed motion. We can calculate the heating of the particles in the trap using the methods described in the literature[S9]. The excess temperature of the environment, ΔT_{NP}, around a nanosphere trapped in water near the glass coverslip is defined as $\Delta T_{NP} = \sigma_{abs} I / 4\pi R \kappa_{glass}$, where σ_{abs} is the absorption cross section of the nanoparticles (1.96×10^3 nm^2 and 3.09×10^3 nm^2 for the 150 nm and 200 nm diameter nanoparticles respectively), I is the incident laser intensity ($1.5\,\mathrm{MW\,cm^{-2}}$), R is the particle radius and κ is the thermal permittivity of glass ($1.4\,\mathrm{W\,(m\cdot K)^{-1}}$), which is the dominant avenue for heat removal in our system. The result of

this calculation is that the temperature difference between the particles is small ($\Delta T_{200nm} = 26.3^0$, $\Delta T_{150nm} = 22.3^0$). This slight temperature difference leads to a slight difference in dynamic viscosity of the water (0.52mPa for the 200nm nanoparticles, 0.56 mPa for the 150 nm diameter nanoparticles). If we consider only water as the sink for thermal energy from the nanoparticles ($\kappa = 0.6\,\mathrm{W\,(m \cdot K)^{-1}}$), the particle temperature will be higher ($\Delta T_{200nm} = 61.3^0$, $\Delta T_{150nm} = 52.0^0$) and the viscosity will be lower (0.32mPa for the 200nm nanoparticles, 0.36mPa for the 150 nm diameter nanoparticles). Even in this regime, the temperature and viscosity difference between the two particles is not strikingly large.

In addition, it is not straightforward to consider our system as a Janus particle because the particles are not physically bound to each other and the separation between them changes. In addition, due to their separation ($\sim$ 600 nm), the temperature around each individual particle will be roughly uniform. The reason for this is that the gap between the particles is significantly larger than their individual size and thus the medium directly around them will be affected, to first approximation, by the heating of the individual particles (see treatment of particle pairs in G. Baffou et al[S9].

Nevertheless, even if we take the temperature difference between the particles as the cause of the driven motion, the observed motion direction is the *opposite* to what is expected for a Janus particle in water. The driven motion we have observed is towards the large particle, which is the slightly warmer particle and experiences a smaller local viscosity. In other words, based on the observed motion the Soret coefficient of our system (defined as $S_T = D_T/D$, where D is the diffusion coefficient and D_T is the thermodiffusion coefficient) is negative. By contrast, previous papers reported a positive Soret constant (*e.g.* motion of the Janus particle towards the cooler side) when the particle was placed in water[S8,S10,S11]. While it is possible to obtain a negative Soret coefficient by adding a surfactant to the water or by decreasing the water temperature, we performed our experiment in pure DI water and at room temperatures, and we do not anticipate a negative Soret coefficient.

Thus, the nature of the motion that we observe – the direction of the dimer motion

towards the larger, hotter particle, and the dependence of the driven motion direction on interparticle separation – suggest that the driving force is not thermophoretic in nature. We conclude that the reason for the driven motion is the electrodynamic interaction between the particles, in agreement with previous theoretical work and with our simulations.

Simulation methods

Force evaluation through Generalized Mie Theory

The electrodynamic interactions are computed using the Generalized Mie Theory (GMT) method.[S12,S13] In GMT, the incident and scattered fields are expanded into the vector spherical harmonic (VSH) functions for each particle. The incident field is expanded into the regular VSH's $\boldsymbol{N}_{nm}^{(1)}$ and $\boldsymbol{M}_{nm}^{(1)}$,

$$\boldsymbol{E}_{\text{inc}}^{j} = -\sum_{n=1}^{L_{\max}} \sum_{m=-n}^{n} \mathrm{i} E_{mn} \left[p_{mn}^{j} \boldsymbol{N}_{nm}^{(1)} + q_{mn}^{j} \boldsymbol{M}_{mn}^{(1)} \right] \tag{S4}$$

where $L_{\max}$ is the maximum number of multipole orders to expand in, E_{mn} is a normalization constant, and p_{mn} and q_{mn} are the expansion coefficients to be solved for. The scattered field is expanded into the scattering VSH's $\boldsymbol{N}_{nm}^{(3)}$ and $\boldsymbol{M}_{nm}^{(3)}$,

$$\boldsymbol{E}_{\text{scat}}^{j} = -\sum_{n=1}^{L_{\max}} \sum_{m=-n}^{n} \mathrm{i} E_{mn} \left[a_{n}^{j} p_{mn}^{j} \boldsymbol{N}_{nm}^{(3)} + b_{n}^{j} q_{mn}^{j} \boldsymbol{M}_{mn}^{(3)} \right] \tag{S5}$$

where a_n^j and b_n^j are the ordinary Mie coefficients[S14] of particle j.

The expansion coefficients are solved for a system of $2NL_{\max}(L_{\max}+2)$ equations,

$$\begin{aligned} p_{mn}^{j} &= p_{mn}^{(j\to j)} - \sum_{l\neq j}^{(1,N)} \sum_{v=1}^{L_{\max}} \sum_{u=-v}^{v} A_{mn}^{uv}(l\to j) a_{v}^{l} p_{uv}^{l} + B_{mn}^{uv}(l\to j) b_{v}^{l} q_{uv}^{l} \\ q_{mn}^{j} &= q_{mn}^{(j\to j)} - \sum_{l\neq j}^{(1,N)} \sum_{v=1}^{L_{\max}} \sum_{u=-v}^{v} B_{mn}^{uv}(l\to j) a_{v}^{l} p_{uv}^{l} + A_{mn}^{uv}(l\to j) b_{v}^{l} q_{uv}^{l} \end{aligned} \tag{S6}$$

where $p_{mn}^{(j\to j)}$ and $q_{mn}^{(j\to j)}$ are the expansion coefficients of the incident source and $A_{mn}^{uv}(l \to j)$ and $A_{mn}^{uv}(l \to j)$ are VSH translation coefficients from particle l to particle j. Solving this system includes induced dipole interactions as well as many-body interaction terms.

Once the expansion coefficients are solved for, the force on each particle can be determined by integrating the Maxwell stress tensor (MST) $\boldsymbol{T}$ over the surface of each sphere,

$$\boldsymbol{F} = \oint_{\Omega} \boldsymbol{T} \cdot d\boldsymbol{\Omega} \tag{S7}$$

Figure S7 shows the angular scattering from a heterodimer with the indicated separations. The small triangles represent the centroid of the angular field distributions showing that the centroid shifts to the $-x$ or $+x$ direction depending on the interparticle separation. That is, the light scattering is asymmetrical due to the asymmetry of the heterodimer and its separation.

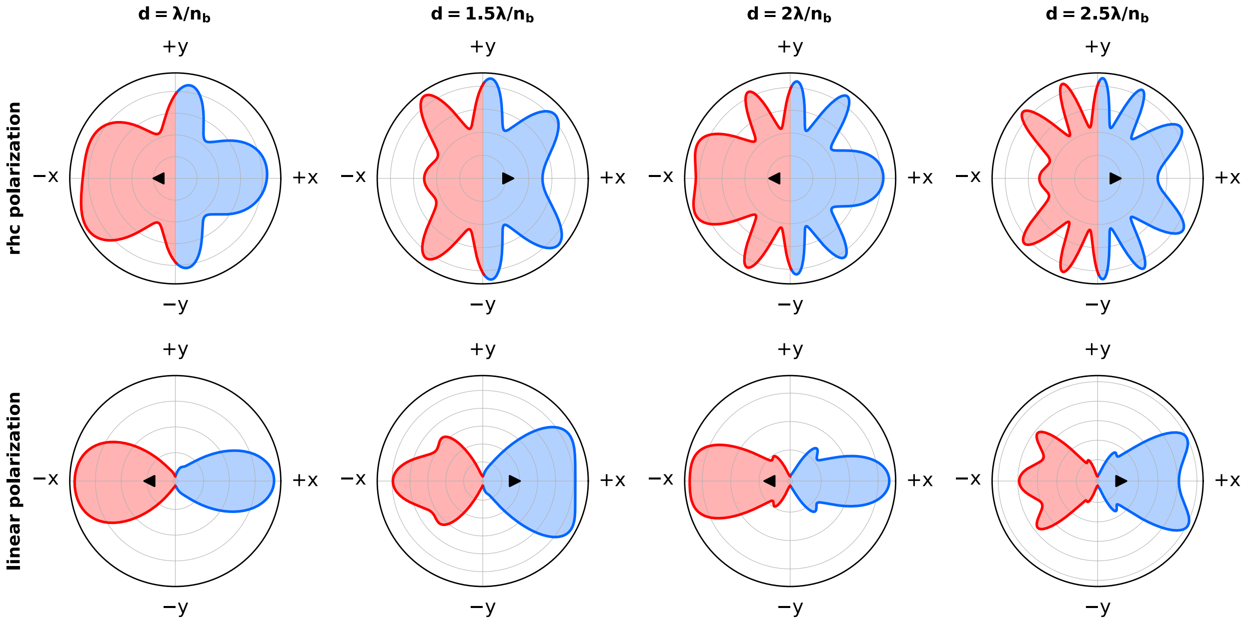

Figure S7: Angular scattering in the xy-plane for the hetero-dimer for different particle separations (integer and half integer multiples of the wavelength) and incident light polarization (right hand circularly polarized and linearly polarized along the y-axis). The black triangle is the centroid of the angular distribution and indicates the preferred direction of angular scattering. For separations equal to an integer multiple of the wavelength, more light is scattered in the $-x$ direction while for half integer multiples more light is scattered in the $+x$ direction.

Langevin equation of motion

The equation of motion for a 2-particle system undergoing dissipation and thermal noise is given by the Langevin equation

$$m_i \frac{d^2 \boldsymbol{r}_i}{dt^2} = \boldsymbol{F}_i(\boldsymbol{r}_i, t) - \lambda_i \frac{d\boldsymbol{r}_i}{dt} + \boldsymbol{\eta}_i \tag{S8}$$

where m_i is the mass of each nanoparticle, $\boldsymbol{F}_i$ is the electrodynamic force on each particle, $\lambda_i = 6\pi\nu R_i$ is the friction coefficient (ν is the dynamic viscosity of water), and $\boldsymbol{\eta}_i$ is a Gaussian noise term such that the fluctuation-dissipation theorem holds. Equation (S8) is integrated in time using a leap-frog Verlet integrator[S15] to give the trajectories of the nanoparticles.

Figure S8 shows the results of GMT-LD simulations of a 150-100nm diameter Ag dimer

pair in a medium with the viscosity of water at T=300 K. The particles initially move to a separation of ∼ 600 nm (optical binding) and then the optically bound hetero-dimer moves as a "rigid body" towards the larger particle as in experiment. The slight wiggles in the trajectory reflect the Brownian (thermal) noise in the LD simulation.

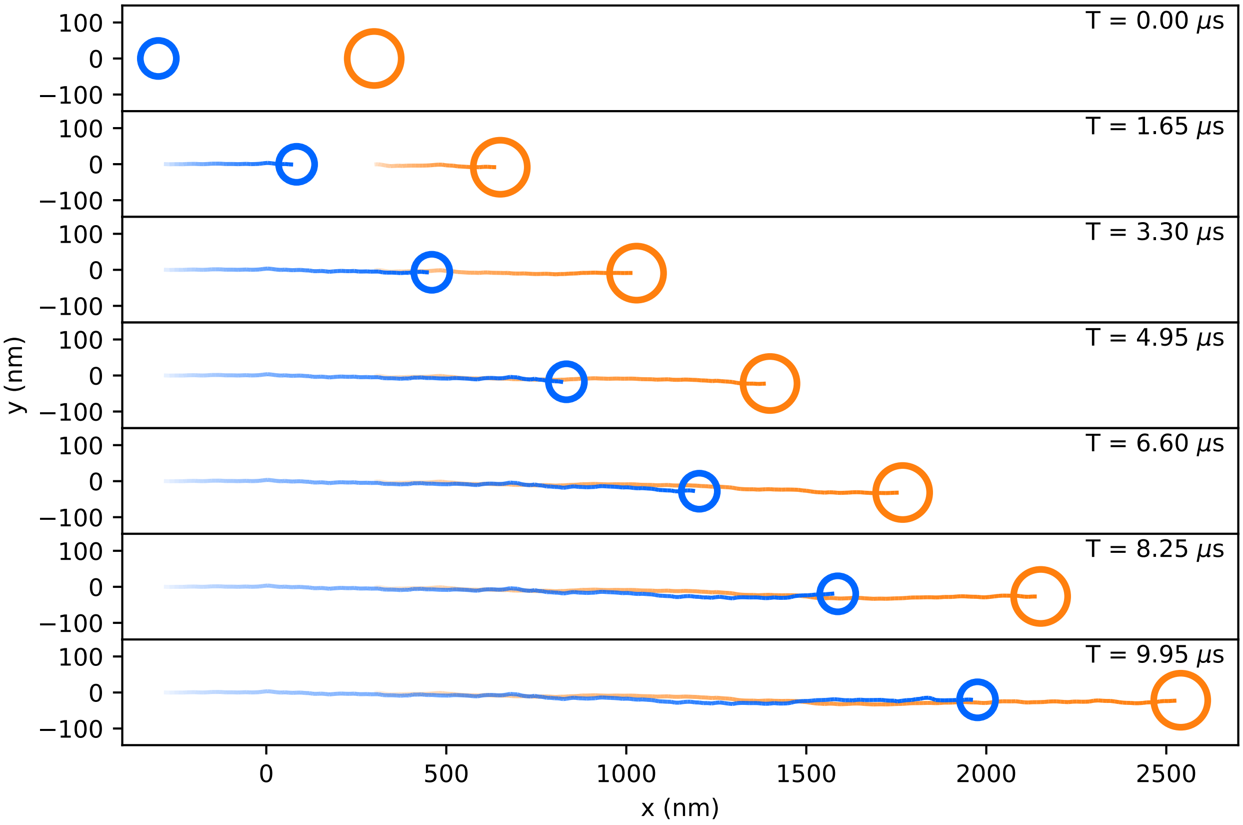

Figure S8: Trajectory snapshots of the simulated hetero-dimer using the GMT-LD method.[S16] The incident source is a y-polarized plane wave with no component of the Poynting vector in the xy-plane. The blue particle is 100 nm in diameter while the orange particle is 150 nm in diameter. A temperature of T = 300 K is used in a water medium (index of refraction n_b = 1.33). The motion of the hetero-dimer is a manifestation of the non-zero (non-reciprical) net electrodynamic force.

Characteristics of Au nanostars

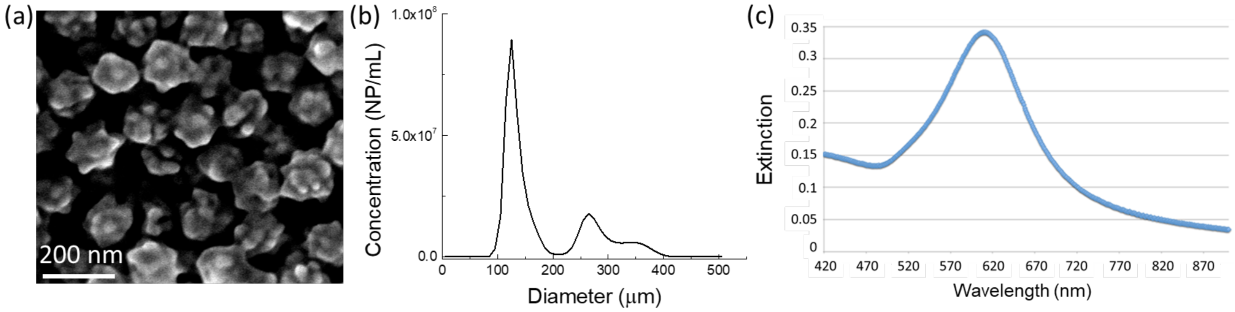

Figure S9: Characteristics of the gold nanostars. (a) Scanning electron microscopy image (SEM) images of Au nanostars. (b) Nanoparticle sizes were determined by tracking analysis (with Nanosight NS300-Malvern) reveal one major peak at 125nm diameter corresponding to the average diameter of single particles, and peaks at 265nm and 350nm. The latter reveals the significant presence of dimer and trimer aggregates in the solution. (c) Extinction spectra of the Au nanostar solution.

Characteristics of Au nanoparticle cluster

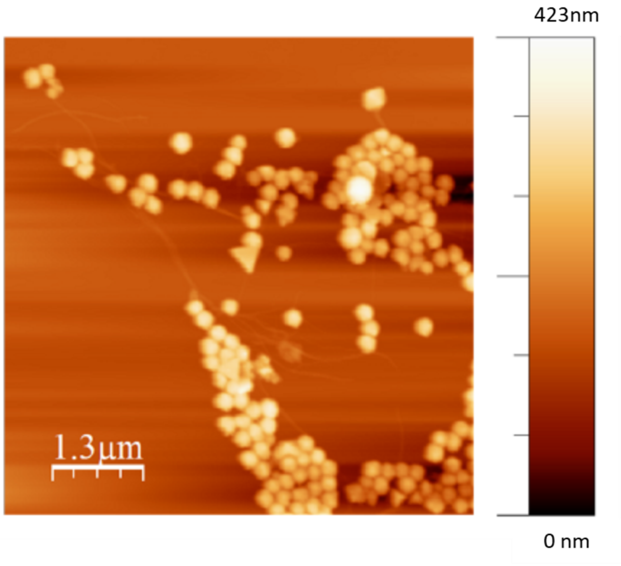

Figure S10: Characteristics of the Au nanospheres that form the large aggregate shown in Figure 4 of the main text. AFM image of the gold nanoparticles of 200nm diameter. Individual Au nanospheres as shown here are also present in the ring trap of Figure 4.

Driven motion of Au nano-star dimer and Au NP aggregate

While circularly polarized light provides isotropic excitation anywhere within the ring trap, we expect that the nanostar particles would spin (and a dimer would rotate) as has been demonstrated for an anisotropic nanoparticle such as a nanorod or nanowire, or nanosphere dimer in the near field interaction regime[S17,S18]. However, the confinement of the ring trap would hinder rigid-body type rotation. Therefore, since we want to focus on linear driven motion, it is desirable to prevent the emergence of a confounding effect such as spinning; even though understanding it will be an interesting separate study. Under linearly polarized light, the isotropic optical field-dimer interaction within the ring trap is broken. We expect the particles to align with the light polarization to minimize the induced torque, and to be driven due to asymmetric scattering. However, as mentioned above, the dimer cannot rotate as a rigid body, but thermal energy could cause internal rearrangements that essentially reverse the direction of the anisotropic polarizability causing a reversal in the direction of light scattering and of its motion. We observe that the dimer spends more time around $\Theta_c = 270^0$ (with an orientation parallel to the polarization). The nanostar dimer is able to rearrange because of the Brownian thermal noise.

We also studied the dynamics of an asymmetrical aggregate of spherical NPs. There is a noticeable difference in the dynamics of the Au NP aggregate vs. that of the nanostar dimer. We believe this to result from the intrinsic scattering properties of the aggregate and the electrodynamic interactions between the aggregate and the many Au NPs present in the ring trap. In the trajectory shown from A to B in Figure 4e in the main text, the mean speed is as high as $384^o s^{-1}$ then decreases to $68^o s^{-1}$ from B to C and increases, after flipping orientation, up to $267^o s^{-1}$ between C and D. Furthermore, as shown in Figure S11, the aggregate is oriented perpendicular (near 270°) and parallel (near 180°) to the polarization before it flips. Several frames from the video that demonstrate this change in orientation

are shown in Figure S11 for a location of the aggregate near 180° in the ring. The speed of the cluster is non-linear, it decreases near 245° which corresponds to the region where it electrodynamically “contacts” the many single Au NPs also trapped in the ring. By contrast, these interactions are negligible in the case of the nanostar dimer because of the lower particle density in the ring.

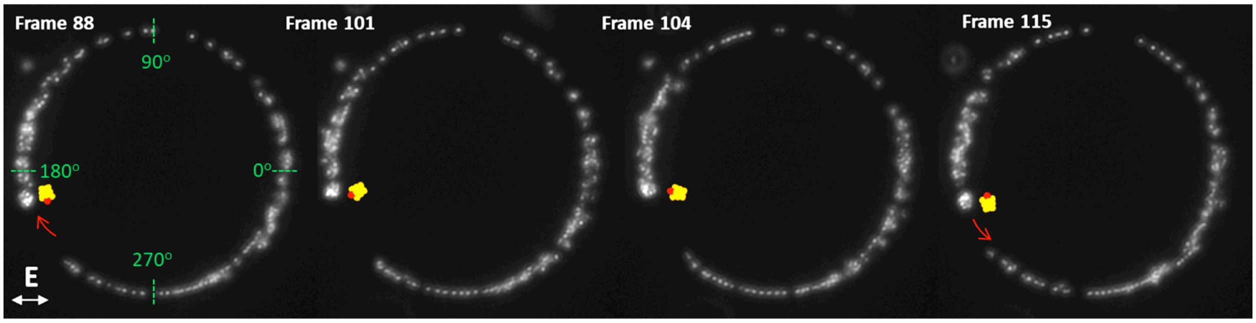

Figure S11: Dark field images of the Au NP aggregate and Au NPs optically trapped in the ring. Images are presented in chronological order showing the evolution of the cluster orientation around 200°. Frame rate is 35fps. The dense groups of individual Au nanoparticles in the $< 180^0$ and $> 270^0$ regimes are due to a slight astigmatism.

The difference in the dynamics between the dimer and the aggregate is thus not only due the intrinsic scattering properties of the aggregates but also the result of the interaction of the aggregate with the optically bound Au NPs in the ring trap. The interactions reduce the net drift force even though the behavior of the aggregate is strongly super diffusive (see Figure 4f). Conversely, the interaction and driven motion of the Au NP aggregate affect the local NP density. As observed in the video, the Au cluster pushes the many single Au NPs inducing a compression of Au NPs in these two regions (around 180° and 285°). Notably, the aggregate does not proceed further presumably both because of the resistance of the Au NPs to further compression and also its interaction with the linearly polarized beam. We beleive the combination of factors cause the Au NP aggregate to rotate 180° at these "turning points" and to then undergo the driven motion in the reverse direction until reaching the other turning piing.

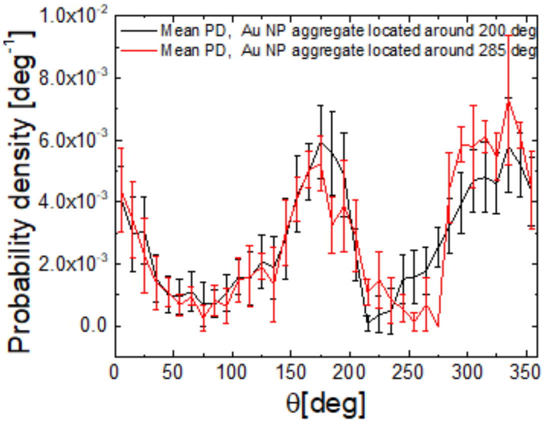

Figure S12: Evolution of the Au nanoparticle (NP) density in the ring. We show the average NP density depending on the two extreme positions of the aggregate in the ring trap. The local NP density is increased near the Au NP aggregate (i.e. near the 200° and 270° location). The error bars represent the standard deviation of the NP density.The Au NP density increases at angular values beyond where the Au NP aggregate goes due to its "sweeping" and forcing them into more compact angular regions of the ring.

As shown in Figure S12, our interpretation is supported by the average probability density calculated for the extreme position of the cluster. At 200° (respectively 285°), there is an increase (respectively a decrease) of the density of NPs around 180° and a decrease (respectively an increase) of the density around 270° when the Au NP aggregate is at 200° (285°) positions. Further investigation of the phenomenon is beyond the scope of the paper.

List of videos

Video S1 - video of homodimer in ring trap.

Video S2 - video of heterodimer in ring trap - motion in a CW direction

Video S3 - video of heterodimer in ring trap - motion in a CCW direction

Video S4 - video of the nanostar dimer in a ring trap.

Video S5 - video of Au nanoparticle cluster in the ring trap.

References

(S1) Horvath, H. *Journal of Quantitative Spectroscopy and Radiative Transfer* **2009**, *110*, 787–799.

(S2) Bastus, N. G.; Merkoci, F.; Piella, J.; Puntes, V. *Chemistry of Materials* **2014**, *26*, 2836–2846.

(S3) Dholakia, K.; Zemánek, P. *Reviews of Modern Physics* **2010**, *82*, 1767–1791.

(S4) Sukhov, S.; Shalin, A.; Haefner, D.; Dogariu, A. *Optics Express* **2015**, *23*, 247.

(S5) Sbalzarini, I. F.; Koumoutsakos, P. *Journal of structural biology* **2005**, *151*, 182–195.

(S6) Figliozzi, P.; Sule, N.; Yan, Z.; Bao, Y.; Burov, S.; Gray, S. K.; Rice, S. A.; Vaikuntanathan, S.; Scherer, N. F. *Physical Review E* **2017**, *95*, 022604.

(S7) Golestanian, R.; Liverpool, T.; Ajdari, A. *New Journal of Physics* **2007**, *9*, 126.

(S8) Jiang, H.-R.; Yoshinaga, N.; Sano, M. *Physical Review Letters* **2010**, *105*, 268302.

(S9) Baffou, G.; Quidant, R.; García de Abajo, F. J. *ACS Nano* **2010**, *4*, 709–716.

(S10) Duhr, S.; Braun, D. *Proceedings of the National Academy of Sciences* **2006**, *103*, 19678–19682.

(S11) Piazza, R.; Parola, A. *Journal of Physics: Condensed Matter* **2008**, *20*, 153102.

(S12) Xu, Y.-l. *Applied Optics* **1995**, *34*, 4573–4588.

(S13) Ng, J.; Lin, Z.; Chan, C.; Sheng, P. *Physical Review B* **2005**, *72*, 085130.

(S14) Bohren, C. F.; Huffman, D. R. *Absorption and scattering of light by small particles*; John Wiley & Sons, 2008.

(S15) Birdsall, C. K.; Langdon, A. B. *Plasma physics via computer simulation*; CRC Press, 2004.

(S16) Sule, N.; Rice, S.; Gray, S.; Scherer, N. *Optics Express* **2015**, *23*, 29978–29992.

(S17) Sule, N.; Yifat, Y.; Gray, S. K.; Scherer, N. F. *Nano letters* **2017**, *17*, 6548–6556.

(S18) Shao, L.; Käll, M. *Advanced Functional Materials* **2018**, 1706272.
